# The Role of the Medical Profession in Combating the Social Evil

**Published:** 1907-02

**Authors:** 


### THE ROLE OF THE MEDICAL PROFESSION IN COMBATING THE SOCIAL EVIL.

Anders (Medicine, Nov., 1906) says the most effective work in improving public morals is to be accomplished through organized effort, in which the medical profession must bear the "heat and burden of the day" in the matter of investigating existing conditions and indicating the means that promise to minimize, and finally to eradicate, if possible, this well-known evil. The profession should adopt a general plan of action if it would hope to execute a winning crusade against evil. The author suggests the following skeletal outline of such a plan: 1. A campaign of education which is to be extend primarily to the mass of the medical profession; this should embrace (a) agitation through co-operative effort on the part of high-class journals, medical writers, and medical organizations, with a view to giving enlightenment to that profession, and (b) the proper training of medical students not only in the widespread significance of the physical ailments resulting from moral indulgencies, but also the moral responsibility involved in this gravely menacing evil. 2. The education by the medical profession of the people, more particularly the rising generation, either directly or with the aid of the parents. 3. The medical profession should invite the co-operation of sanitarians, statisticians, philanthropists and public spirited citizens in general. In order to effectively carry out this work a perfect and complete organization of professional and lay forces animated by a unity of purpose and action is absolutely essential. 5. Governmental supervision, which should partake of a sort of parental care or oversight, and special municipal regulations, are to be recognized as auxiliary agencies of considerable value in suppressing social evil.

